# Chiropractic services in the active duty military setting: a scoping review

**DOI:** 10.1186/s12998-019-0259-6

**Published:** 2019-07-15

**Authors:** Silvano Mior, Deborah Sutton, Daphne To, Carolina Cancelliere, Simon French, Anne Taylor-Vaisey, Pierre Côté

**Affiliations:** 1 UOIT-CMCC Centre for Disability Prevention and Rehabilitation, University of Ontario Institute of Technology (UOIT) and Canadian Memorial Chiropractic College (CMCC), 6100 Leslie Street, Toronto, Ontario M2H 3J1 Canada; 2 Department of Research and Innovation, Canadian Memorial Chiropractic College, 6100 Leslie Street, Toronto, Ontario M2H 3J1 Canada; 3 Department of Graduate Studies, Canadian Memorial Chiropractic College, 6100 Leslie Street, Toronto, Ontario M2H 3J1 Canada; 4 Faculty of Health Sciences, University of Ontario Institute of Technology (UOIT), 2000 Simcoe Street North, Oshawa, Ontario L1G 0C5 Canada; 5 Department of Chiropractic, Faculty of Science and Engineering, Macquarie University, Level 3, 17 Wally’s Walk, North Ryde, NSW 2109 Australia; 6 Faculty of Health Sciences, University of Ontario Institute of Technology (UOIT), 2000 Simcoe Street North, Oshawa, Ontario L1G 0C5 Canada

**Keywords:** Military personnel, Active duty, Chiropractic, Military medicine

## Abstract

**Background:**

Musculoskeletal injuries are one of the most prevalent battle and non-battle related injuries in the active duty military. In some countries, chiropractic services are accessed to manage such injuries within and outside military healthcare systems; however, there is no recent description of such access nor outcomes. This scoping review aimed to synthesize published literature exploring the nature, models, and outcomes of chiropractic services provided to active duty military globally.

**Method:**

We employed scoping review methodology. Systematic searches of relevant databases, including military collections and hand searches were conducted from inception to October 22, 2018. We included peer-reviewed English literature with qualitative and quantitative designs, describing chiropractic practice and services delivered to active duty military worldwide. Paired reviewers independently reviewed all citations and articles using a two-phase screening process. Data from relevant articles were extracted into evidence tables and sorted by study type. Results were descriptively analyzed.

**Results:**

We screened 497 articles and 20 met inclusion criteria. Chiropractic services were commonly provided on-base only in the US. Services were accessed by physician referral and commonly after initiation or non-response to other care. Use of scope of practice was determined by the system/facility, varying from intervention specific to comprehensive services. Back pain with and without radiculopathy accounted for most complaints. Treatment outcomes were reported primarily by case reports. However, two recent randomized trials reported improved pain, disability, and satisfaction when adding chiropractic care to usual medical care compared to usual medical care alone in management of low back pain. Specific reaction time measures in special operation forces military did not improve after chiropractic care compared to wait-list control.

**Conclusions:**

Our scoping review found the majority of published articles described chiropractic services in the active duty military in the US setting. Recent RCTs suggest a benefit of including chiropractic care to usual medical care in managing back pain in active duty military. Yet despite reported benefits in Australia, Canada, and the US, there is a need for further qualitative, descriptive, and clinical trial data worldwide to inform the role of chiropractic services in active duty military.

**Electronic supplementary material:**

The online version of this article (10.1186/s12998-019-0259-6) contains supplementary material, which is available to authorized users.

## Background

Musculoskeletal injuries significantly affect the health and operational readiness of active military personnel. They are one of the most prevalent battle and non-battle related injuries in theatre [1, 2]. Analysis of United States (US) Navy Physical Evaluation Board data between February 2005 and February 2006 indicated that musculoskeletal diagnoses were frequent (43%), with back pain (29%) being the most common musculoskeletal diagnosis [3]. Musculoskeletal injuries are also one of the most common reasons for Canadian Armed Forces (CAF) personnel not being deployed [4], and were responsible for 42% of all medical releases in 2013 [5]. In addition, neck pain is an important aeromedical problem. Fifty-one percent (51.7%) of Swedish Air Force aviators reported experiencing neck pain [6], while 53.3% of rotary-wing crew and 69% of fast-jet crew reported neck pain in the Royal Air Force [7].

In the active military setting, musculoskeletal conditions are associated with lost productivity due to sick parade attendance, lost duty days [1, 8, 9], and impact the ability to deploy [5]. The probability of returning to full duties decreases with time spent away from duties [10], and the potential long term sequelae include limited duty assignment or early termination of service [8]. In addition to related costs for treatment interventions, medical discharge increases resource expenditures resulting from the recruitment and training of replacement recruits [2].

Evidence-based interventions for musculoskeletal injuries include a focus on active versus passive treatment, structured education, exercise, and manual and cognitive behavioural therapies [11]. Musculoskeletal programs of care in the military setting are frequently delivered in a multidisciplinary healthcare environment. Access to these programs of care by military personnel most often occurs through a traditional gatekeeper physician referral [12], or through a non-traditional gatekeeper such as a physical therapist [13]; personnel are referred to other musculoskeletal healthcare providers, or to team assessment and management [14, 15].

Available reviews have compared or described chiropractic services within both military and veteran healthcare systems in combination [16–18]. A 2009 review described chiropractic services in military and veteran healthcare systems in the US and Canada, but concluded that there was a need to evaluate the processes, policies, practices, and effectiveness of chiropractic services in these settings [17]. However, no recent knowledge syntheses have summarized the integration of chiropractic services in global military healthcare systems solely within an active duty military population. In particular, to our knowledge there is no current review of the literature describing chiropractic services and its utilization, scope of practice, and policies in the active duty military worldwide. Such a review can assist in informing the role of chiropractic services in this population.

Therefore, the objective of this scoping review was to document the current global state of knowledge related to chiropractic services in the active duty military setting with respect to: 1) access of chiropractic services; 2) chiropractic scope of practice, e.g. procedures, processes, and actions; 3) service model and location; and 4) type of condition treated, duration, and outcomes of treatment provided to active duty military members.

## Methods

We employed scoping review methodology to collect and organize relevant information to synthesize the available evidence addressing our broad research question [19]. We applied the scoping review framework of Arksey and O’Malley [19] and successive recommendations [20–22] for conducting and reporting scoping reviews. Consistent with this framework, we did not critically appraise the methodology of reviewed articles [19–21]. This review is reported against the PRISMA extension for scoping reviews (PRISMA-ScR) [22].

### Stage 1: identifying the research question

Our scoping review was guided by the following broad research question: *What is published in the peer-reviewed literature regarding the access, scope of practice, service models, conditions treated and outcomes related to chiropractic care for active duty military members?*

### Stage 2: identifying relevant articles

Our search strategy was developed in consultation with a health sciences librarian, and a second librarian reviewed the search for completeness using the Peer Review of Electronic Search Strategies (PRESS) Checklist [23, 24]. The search strategy was first developed in MEDLINE (Ovid®) and subsequently adapted to the other databases. The search terms included subject headings specific to each database (e.g., MeSH in MEDLINE) [25] and free text words relevant to utilization of chiropractors and chiropractic services were combined with terms relevant to the army and active duty military (see Additional file 1 for full MEDLINE search strategy).

**Fig. 1:**
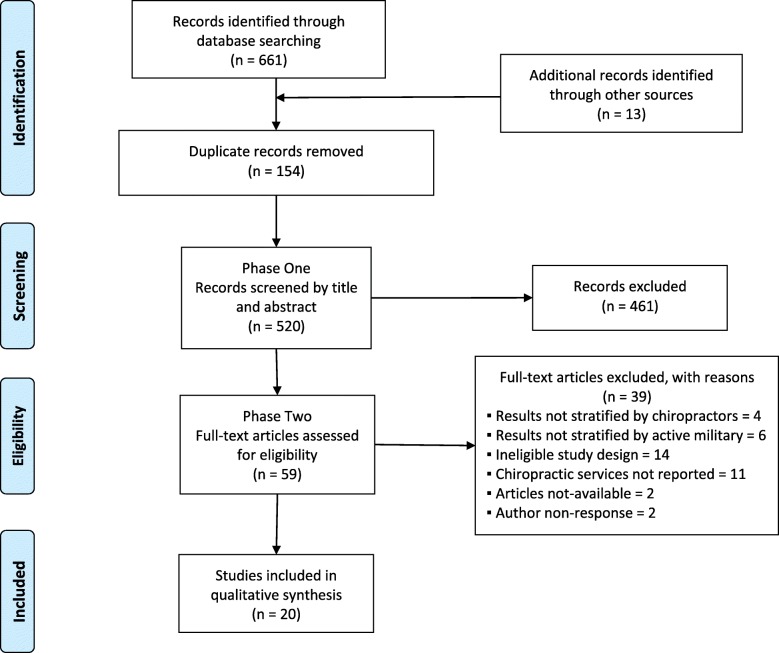
PRISMA-ScR (PRISMA extension for Scoping Reviews

We searched MEDLINE, Ovid MEDLINE In-Process and Other Non-Indexed Citations, PsycINFO, Cochrane Central Register of Controlled Trials, and Embase, through Ovid Technologies, Inc.; CINAHL Plus through EBSCOhost from inception to September 15, 2018; and the Military & Government Collection through EBSCOhost; and the Military Database through ProQuest from inception to October 22, 2018. The reference lists of relevant articles were hand searched for additional articles not identified from the electronic database search. We used the PRISMA-ScR [22] flow chart to track the number of articles at each stage of the review. The results from the database searches were combined and imported to EndNote X6 [26]. We did not register this review prior to undertaking it.

### Stage 3: article selection

#### Inclusion and exclusion criteria

Eligible studies met the following criteria: 1) published in the peer-reviewed literature; 2) written in the English language; 3) were any primary qualitative or quantitative designs, including qualitative studies, randomized controlled trials, quasi-randomized trials, cohort, cross-sectional, case report and case series designs; 4) described chiropractic services; and 5) the study population included active duty military personnel, the National Guard, or reservists. Study exclusion criteria included: narrative and systematic reviews, letters, editorials, commentaries, unpublished manuscripts, dissertations, government reports, books and book chapters, conference proceedings, meeting abstracts, lectures and addresses, consensus development statements, informal communication, e.g. blogs, podcasts, email, cadaveric or animal studies, and non-active military members, e.g. veterans.

#### Screening and agreement

Eligible articles were selected through a two-phase screening process. In Phase 1, two of the authors (DS, DT) independently screened titles and abstracts to determine eligibility. Articles were classified as relevant, possibly relevant, or irrelevant. In Phase 2, the same reviewers independently reviewed full text manuscripts of relevant and possibly relevant articles to make a final determination of eligibility. Reviewers met to solve disagreements and reach consensus in both phases. We involved a third independent reviewer (SM) if consensus could not be reached. We contacted authors when additional information was needed to confirm article relevance.

### Stage 4: data charting

We extracted the following data from the relevant articles (when available): 1) study description (study design, country of origin, service model and branch, and study population); 2) type of condition and duration; 3) chiropractic services provided; and 4) study findings (e.g. utilization of chiropractic services, patient outcomes, satisfaction). One review author (DS) extracted the data which were independently checked by a second review author (SM) to minimize error.

### Stage 5: collating, summarizing, and reporting the results

We employed a ‘descriptive-analytical’ method within the narrative tradition to summarize the data and include the following [27]:

1. Descriptive numerical analysis: The nature and distribution of the articles were examined with respect to the total number of articles, year of publication, country where studies were conducted, study population, and study design.
2. Narrative summary of included study findings: We classified the studies according to our review objectives: 1) access to chiropractic services; 2) chiropractic scope of practice, e.g. procedures, processes, and actions; 3) service model and location of delivery; and 4) type of condition treated, duration and outcomes of treatment provided for active duty military members globally. Where relevant and where possible, we extracted the 95% confidence intervals around any point estimates provided.
3. Implication of results: We reported the findings according to our objective of describing the published literature on utilization, scope of practice, and policies related to chiropractic services for active duty military members globally.

## Results

Our search yielded 674 citations. We removed 154 duplicates and screened 520 articles (Fig. 1). During Phase I screening, we excluded 461 articles, and a further 39 articles following Phase II screening. We contacted two authors for clarification, one regarding military status of participants and one to clarify treatment specifics of chiropractic care provided. However, neither author responded; these two articles were excluded. Twenty articles were included in this review [12, 16, 28–45].

### Descriptive numerical analysis

Table 1 summarizes the key findings from the relevant articles. Research on the utilization, scope of practice, and policies related to chiropractic services for active duty military members globally is a small and relatively recent body of literature, with the earliest included study published in 2006. The studies were most commonly conducted in the United States (*n* = 17) [16, 28–32, 34–43, 45], with one conducted in Australia (*n* = 1) [33], and two in Canada (*n* = 2) [12, 44]. Most studies were cross-sectional (*n* = 9) [12, 28, 31–37], six were case reports [38–43], three were randomized controlled trials [29, 30, 45], and two were qualitative designs [16, 44].

**Table 1:** Summary of location, population, condition of interest and key findings reported in included studies (*n* = 20)

First Author, Year	Country, Service Branch, Service Location,	Population, Number, Groups	Condition and Duration	Treatment	Key Findings: visit-specific information, outcomes, adverse events
	Randomized Control Trials				
DeVocht 2019 [45]	United States Blanchfield Army Community Hospital, KY SOF	Personnel On-base	Little or no body pain (avg pain intensity < 4 on 10 scale)	CMT: HVLA SMT to cervical, thoracic, lumbopelvic areas, as indicated	Mean age 33 ± 5.6 years; male 100%
		CMT 4 visits over 2 weeks (*n* = 60) vs Wait-list Control (*n* = 60)			Pain intensity: median (range): 2.0 (0–3.0)
					Primary Outcome: Mean change (95% CI) between CMT and wait-list control at 2 weeks not statistically significant:
				Wait-list Control: no treatment	
					Hand simple reaction time: −3.49 (−24.75 to 18.77)
					Foot simple reaction time: 0.97 (−18.04 to 19.98)
					Choice reaction time: 3.49 (− 14.40 to 21.39)
					Fitt’s Law test response time: 0.99 (−0.37 to 2.35)
					t-wall response time: − 0.41 (−1.24 to 0.41)
					Secondary Outcome:
					Mean change (95% CI) pre- and post-reaction response time at visit 2 and final visit in favor of CMT for t-wall response time only.
					Visit 2 t-wall response time:-0.90 (−1.71 to −0.09)
					Final Visit t-wall response time: − 0.75 (−1.43 to − 0.06)
					Adverse events: 0 related to trial procedures 4 related to activities
Goertz, 2013 [29]	United States	On-base personnel	Acute LBP (<4wks)	CMT: including HVLA SMT, massage, exercises, McKenzie exercises, mobilization, advice-ADL, postural, ergonomic	Mean age 26 years; male 86%
					Mean duration of complaint 9 days
					Radicular signs in 43% of participants
					Mean visits SMC 1.4; mean SMC 1 + CMT 7
					Mean difference favouring SMC + CMT at 2 weeks:
				SMC: include usual care, medications, physical therapy, pain clinic	
					RMDQ 3.9 (95%CI 1.8, 6.1);
					NRS 1.2 (95%CI 0.2, 2.3);
					BPFS −7.7 (95%CI −12.9, − 2.6
					Mean difference favouring SMC + CMT at 4 weeks:
				2 visits weekly over 4 weeks	
					RMDQ 4.0 (95%CI 1.3, 6.7);
					NRS 2.2 (95%CI 1.2, 3.1);
					BPFS −10 (95%CI − 14.6, −5.5)
					SMC vs SMC + CMT satisfaction with care (mean) at Week 2 = 4.5 and 8.9 and at Week 4, 5.4 and 8.9, respectively
					Global Improvement (% moderately better to completely gone): SMC 17%; SMC + CMT 73%
	Army				
	William Beaumont Army Medical Center	SMC + CMT – 2 visits/wk. over 4 wks (*n* = 45) vs SMC (*n* = 46)			
					Participants had higher expectation of helpfulness with SMC + CMT
					No follow-up assessments: SMC 35%; SMC + CMT 15%
					No serious adverse events. Two mild, expected events reported in SMC + CMT group – 1 unrelated to intervention, 1 sharp pain in LB, referred for medication and resolved in 48 h.
Goertz, 2018 [30]	United States	Active duty service personnel	LBP (any duration)	UMC with	Mean age 30.9 (8.7) years; 23.3% female
	Army, Navy			Chiropractic Care: UMC plus up to 12 visits of chiropractic care including SMT, rehabilitative exercise, interferential current; ultrasound, cryotherapy, superficial heat, other manual therapies	Mean visits UMC with at least 1 visit to UMC clinician: Walter Reed 2.6 (2.3); San Diego 2.7(2.5); Pensacola 2.3 (2.3)
	Walter Reed National Military Medical Centre, Naval Medical Centre San Diego, Naval Hospital Pensacola	UMC with chiropractic care (*n* = 375) 12 visits over 6 wks vs UMC (*n* = 375)			
					Mean visits UMC with chiropractic care with at least 1 visit to UMC clinician:
					Walter Reed 2.6 (3.1); San Diego 3.5 (3.0); Pensacola 1.6 (1.6)
					Mean visits to chiropractor with at least 1 chiropractic visit:
					Walter Reed 4.7 (2.5);San Diego 2.3 (1.4); Pensacola 5.4 (2.6)
				UMC: include self-management advice, pharmacologic pain management, physical therapy, pain clinic referral	
					Mean duration (months)
					UMC:
					< 1144 (38.4)
					1–3 40 (10.7)
					> 3191 (50.9)
					UMC with chiropractic care:
					< 1143 (38.1)
					1–3 39 (10.4)
					> 3193 (51.5)
					Primary Outcomes:
					Differences observed at all 3 sites
					Mean difference favoring UMC with chiropractic care at 6 weeks:
					NRS: −1.1 (95% CI −1.4 to −0.7)
					RMDQ: −2.2 (95% CI −3.1 to −1.2)
					Mean difference favoring UMC
					with chiropractic care at 12 weeks:
					NRS (average): − 0.9 (95%CI − 1.2 to − 0.5
					RMDQ: −2.0 (95% CI −3.0 to − 1.0)
					Secondary Outcomes:
					Differences observed at all 3 sites
					Mean difference favoring UMC with chiropractic care at 6 weeks:
					NRS (worst): −1.2 (95% CI −1.6 to −0.8)
					Bothersomeness: − 0.4 (95% CI − 0.6 to − 0.2)
					Mean difference favoring UMC
					with chiropractic care at 12 weeks:
					NRS (worst): −1.1 (95% CI −1.6 to −0.7)
					Bothersomeness: − 0.4 (95% CI − 0.6 to − 0.2)
					Significantly better global
					perceived improvement favoring UMC with chiropractic care at 6 weeks:Observed at all 3 sites OR 0.18 (95% CI 0.13 to 0.25)
					Significantly greater mean satisfaction with care favoring UMC with chiropractic care at 6 weeks:Observed at all 3 sites2.5 (95% CI 1.6 to 3.0)
					Significantly less pain medication
					use favoring UMC with chiropractic care at: 6 weeks: OR .73 (95% CI 0.54 to 0.97)
					12 weeks: OR 0.76 (95% CI 0.58 to 1.00)
					No serious related adverse events. 62 events reported: UMC alone – 19 (3 medication related, 4 epidural injections, 12 muscle/joint stiffness physiotherapy or self-care related. UMC + chiropractic care – 43 (37 muscle/joint stiffness related to chiropractic care and 1 related to physiotherapy care, 1 post epidural injection, 3 not treatment specified, 1 lower limb burning sensation 20 min post manipulation.
	Cross-sectional Surveys				
Boudreau, 2006 [12]	Canada	On-base Patients, *n* = 102 Physicians, *n* = 12	MSK complaints	Joint manipulation, soft tissue massage, stretching, exercise	Patients – response rate 68%; mean age 37 yr. (SD 8)
	Navy				
					Presenting complaint: 97% axial MSK complaints (52% LBP), 3% extremities; current episode: 41% acute, 56% chronic;Average visits/patient: 5.7 (SD 4.1); 94.2% were satisfied with chiropractic care
	Archie McCallum Hospital, CFB Stadacona				
				Adjunct treatment: interferential current, acupuncture	100% agreed: office was easy to get to, attending DC treated them
					with respect and concern; 98.6% agree DCs ability to answer questions; 98.5% high satisfaction with clinic hours of operation; 97.1% agreement that DC thought patients were important and was careful to check everything in the examination; 37.6% disagreed or unsure if DC office had appropriate equipment; 33.2% patients reported improvements took longer than expected; and 30.3% expected better results or were unsure if they should have expected better results
					Physicians: 100% perceived demand from patients for DC;80.6% satisfied with DC services
					Reasons for referral: axial MSK complaints, unresponsive to PT, patient request, PT waiting list too long, history of positive response to DC.
Goertz, 2013 [28]	United States, Outside continental United States, Afloat status for Navy	Active duty personnel *n* = 30,664			Response rate 51.8%; 5.2% (0.46 SE) reported using chiropractic in preceding 12 months (male 4.9% (0.44 SE; female 6.9% (0.96 SE)
					ORs of using chiropractic: 30–39 years 2.26 (95% CI 1.08, 4.74) and 40+ years 3.42 (95% CI 1.36, 8.58) more likely than < 29 years; Black/non-Hispanic 0.35 (95% CI 0.19, 0.66) less likely than White/non-Hispanic; 4 year college education 3.36 (95% CI 1.46, 7.72) more likely than high school education
	Army, Navy, Marine Corps, Air Force				
	Stratified sample of 60 military installations by service and world region, including afloat status for Navy				
					Adjusted prevalence of chiropractic use (2005): 6.2% (0.62 SE) is less than NHIS (2002): 7.5% (0.19 SE) or NHIS (2007) 8.6% (0.27 SE)
Herman, 2017 [31]	United States	MTF, *n* = 142			Response rate 94% (133/142) 110 MTFs provided CAM services and 60 (55%) of MTF offer chiropractic services; 5 reasons/conditions for using chiropractic services (*n* = 49): back pain 47 (42.7%), chronic pain 44 (40.0%), headache (excluding TBI related pain) 30 (27.3%), acute pain (post trauma/injury, postop, preop 30 (27.3%), general health/wellness/prevention 12 (10.9%); MHS (2013) number unique patients 55,843; average patient/visits 5.367; average procedures/visit 1.05; MTF estimated number of chiropractic patient encounters 168,00/year
	Air Force, Army, National Capital Region Medical Directorate, Navy and Marine Corps				
	Military treatment facilities				
Jacobson, 2009 [32]	United States	Active duty personnel, *n* = 86,131			Response rate Panel 1 71%; Panel 2 25%
	Air Force, Army, Marine Corps, Navy, Coast Guard, Reserve/National Guard				
					10.5% reported using chiropractic care in the preceding 12 months
Netto, 2011 [33]	Australia	RAAF Air Combat Group *n* = 86			Response rate 95% (82/86)
	Air Force				78% of Royal Australian Air Force Fast Jet Aircrew experienced flight-related neck pain during or after a flight 55% sought treatment for pain; ~ 12% sought chiropractic treatment for flight-related neck pain; ~ 22% reported chiropractic treatment most effective for flight-related neck pain
	Off base chiropractic care, which is accessed on a case-by-case basis usually after the failure of on-base services				
Petri, 2015 [34]	United States	Active duty personnel			Response rate 2005 100%, 2009 92.1% MTFs: chiropractic services available - 2005 (92%) and 2009 (85%); providing individual chiropractic services - 2005 (92%) and 2009 (79%); number of chiropractors 2005 [15] and 2009 [19]
	DoD MTFs surveyed: Army (*n* = 8), Navy (*n* = 3), Air Force (*n* = 2), other (*n* = 1) 2005 (*n* = 14) and 2009 (*n* = 13)				
Ryan, 2007 [35]	United States	Active duty and reserve personnel *n* = 214,338			Response rate 36% (77,047/214,338)
	Army, navy, Coast Guard, Air Force, Marines.				
					Chiropractic care use: Active duty 8.0%; Reserve/Guard 14.8%
Smith, 2008 [36]	United States	Population: n ~ 550,000			Response rate 39% (1446/3683); Results reported on 1310 of 1372 active duty; 8.6% reported using chiropractic care in the preceding 12 months; participants assisted by practitioner with chiropractic services were at increased risk of future hospitalization compared to those self-reporting such use (HR 1.96; 95% CI 1.01, 3.80)
	Navy, Marine Corps, Reserve Navy and Marine Corps				
		In-patient and out-patient			
		Surveyed random sample: *n* = 5000 but 3683 were eligible			
White, 2011 [37]	United States	In-patient and out-patient Surveyed random sample active duty personnel, *n* = 44,287			29% reported using at least one practitioner assisted CAM 8.1% reported using chiropractic care in the preceding 12 months
	Army, Navy, Air Force, Marine Corps, Coast Guard				
	Standard Inpatient Data Record; DoD TRI-CARE Management Activity’s Health Care Service Record, Standard Ambulatory Data Record				
	Case Report				
Green, 2006 [38]	United States	36 yo, male	Acute non-specific LBP	Interdisciplinary treatment, with chiropractic care provided over 16 visits in 30 weeks, included HVLA SMT, mobilization, active myofascial release therapy, exercise, ischemic compression.	Hospitalization for 24 h, confined to quarters for 72 h and not allowed to return to flying until cleared by flight surgeon.
	USMC				
	Air station Hospital				
					Consultation and treatment with physiatrist and PT. PT referred to DC at 4 months.
					Pain free and return to full function 1 month after last chiropractic visit.
Green, 2008 [39]	United States	23 yo, male	LBP (persistent synchondrosis of primary sacral ossification center)	Treatment: HVLA SMT of sacroiliac joints, stretching, conditioning strengthening and exercises, NSAIDs, advice. Frequency: initial treatment – 2 weeks; 6 weeks after consulting GMO further investigation; recommence treatment – 4wks.	Referred to attending chiropractor.
	Marine				
	Naval Medical Center San Diego				Insidious onset after training exercise.
					At baseline: Verbal pain scale 7/10 to 9/10 when severe; RMDQ: 14/24; no neuro deficits
					Discharged and full RTD.
Green, 2010 [41]	United States	Instructor pilot 38 yo male	Uncomplicated mechanical neck pain	Treatment: 4 visits over 5 wk. & f/u at 6 months	Intermittent neck pain related to frequent flying F/A-18
	Naval Medical Center San Diego Marine Corps				
				Included: active stretching, HVLA SMT, stretching and strengthening home exercises;	Referred for chiro care after no change in symptoms with 2 wks acetaminophen
					At baseline: NRS 3/10; NDI 6%; limited end range of motion on right; no neuro deficits
					Resolved and full RTD
Green, 2014 [40]	United States	Helicopter mechanic 29 yo, male	Mechanical cervico-thoracic pain & myalgia	Interdisciplinary treatment	Chronic neck/upper back pain of 7 yrs. post flexion injury with concurrent tinnitus, dizziness and headaches
	Naval Medical Center San Diego Marine				
				Chiropractic care: 8 visits; HVLA SMT, soft tissue mobilizations, advice, home exercises (stretching, strengthening, proprioceptive); Physical therapist care: 5 visits; acupuncture	
					Baseline: VPS 7/10, painful limitation in motion, no neuro deficits, x-rays-DDD, right elongated styloid process, left calcified stylohyoid ligaments
					Treatment discontinued, reported decrease stiffness, VPS 4/10, no adverse events
					Returned to work.
Lillie, 2010 [42]	United States	40 yo, male	Acute episode LBP with radiculopathy	Interdisciplinary treatment, with chiropractic care provided over 11 visits in 72 days. Treatment included HVLA and mechanically assisted SMT, interferential therapy, cryotherapy, moist heat, nutritional and psychosocial advice, exercises.	Returned to regular exercise routine and able to perform all required Navy Physical Readiness Tests.
	Navy Military Treatment Facility Chiropractic Clinic				
					Subjective complaints resolved and full RTD.
Morgan, 2014 [43]	United States	Military officer 25 yo male	C3–5 ALL heterotopic ossification and ankylosis	Interdisciplinary treatment including oxycodone HCL/ acetaminophen; chiropractic care: 1/wk. for 13wks, then 1/wk. for 8wks, 1/2wks for 26wks - HVLA SMT thoracic spine, respiratory therapy, aqua therapy	Traumatic head injury & right femoral fracture from motor vehicle collision 16 months prior
	Walter Reed National Military Medical Center				
					Baseline: neck and upper back, bilateral hip, knee, wrist, and shoulder pain; VPS 3/10; extremely limited range neck motion; restricted neck & thoracic joint motion; decreased respiratory excursion .5 cm; active deep tendon reflexes; increase CRP, ESR, calcium, alkaline phosphate
					Normal chest expansion increased to 3.5 cm, decrease pain
	Qualitative Studies				
First Author, Year	Country, Service Branch, Service Location,	Population, Documents	Key Findings		
Dunn, 2009 [16]	United States	2-option analysis Legislative reports, policy documents, published works	System Related: chiropractic care available at 49 designated MTFs, planned expansion of 11 new locations in 2009–10; TRICARE chiropractic benefit available to active duty service members but not dependents.		
	DoD				
			Legislative History: chiropractic integrated in MHS as result of 10 pieces of legislation enacted over 17 yrs. (1993–2009).		
			Programmatic Growth: initiated as MHS demonstration project (1995); 5-fold increase in number of commands over 14 yr. period.		
			Leadership Structure: In MHS, leadership for chiropractic program at each command at department head or equivalent, usually two levels below hospital commanding officer. Each branch has Specialty Advisor responsible for issues related to chiropractic activities. No chiropractors functioning at DoD leadership levels. Decentralized structure of MHS and lack of chiropractor in leadership could impact integration.		
			Employment Status of Providers: Chiropractors in MHS serve in role of contractor or employee of contractors. Navy contracts directly with chiropractors (typically with no major benefits); Army and Air Force contracts with contracting organizations. Contractual relationships limited by contract period and if employees by contractors contract. Chiropractors in MHS may experience less job security and benefit “growth”.		
			Clinical Work Duties: Chiropractors work within set of parameters (privileges) as established within system/facility, providing comprehensive chiropractic services (e.g. SMT, mobilizations, modalities, rehabilitation), uphold guidelines, and may perform administrative tasks. Typically supervised by non-chiropractor officers. Quality assurance via peer review. Informally, chiropractors interact with other providers in highly transparent environment, attend regular staff meetings, provide in-service training, maintain competencies, and adhere to documentation requirements.		
			Patient Access: Chiropractic care accessed largely by gatekeeper referral, which may act as limiting factor. Patients must be seen within 30 days.		
			Patient Demographics: In DoD, chiropractors care for mix of active duty and active duty veteran patients, most likely for musculoskeletal conditions.		
			Academic Affiliations and Research: First training rotation within DoD in 2001 with New York Chiropractic College at National Naval Medical Center. Two others established but closed. Little research conducted in DoD and no research time provided in contracts.		
Mior, 2018 [44]	Canada	Canadian Forces Health Services Key informant interviews: MD (*n* = 7), PT (*n* = 13), DC (*n* = 5)	Participant perspectives to Barriers, Opportunities and Recommendations to Integrated Chiropractic Services within CFHS: Barriers: 1: Referring to Off-base Chiropractic Services (base-to-base Variation; Gatekeeper Roles; Care Delivery Unit Medical Officer or Lead physiotherapist designated to chiropractor referral role; Decision to refer to chiropractor based on individual clinician preference and experience, rather than a systematic approach).		
	Canadian Armed Forces				
			2: Inter-professional Communication (Communication processes affected by site-specific resources and current practices; Current practices reflect clinician perspective and past experience; Written communication (referral, reports) not standardized; No dialogue between health care providers on base and chiropractors).		
			3. Duplication of Health Care Services (Scope of practice change: physical therapists and chiropractors; Difficulty distinguishing chiropractor as a profession rather than an intervention; Non-uniform personnel, e.g. chiropractor not able to deploy) Opportunities:		
			1. Musculoskeletal Disorders (Prevalence of MSK conditions, provide care which is clinical and cost effective)		
			2: Inter-professional Collaborative Care (Collaborative, integrated, patient-centered care; Base-to-base variation dependent upon location, size, resources and primary purpose; Co-location of providers strengthens inter-professional communication and relationships)		
			3: Evidence-Based Approach (Standardization of clinical care using clinical practice guidelines based upon high quality evidence)		
			4: The Spectrum of Care (Knowledge of CAF spectrum of care; Utilize chiropractors’ full scope of practice)		
			Recommendations:		
			1. First establish personal rather than professional-level relationships		
			2. Explicate role and responsibilities of chiropractor based on scope of		
			practice		
			3. Standardize communication and treatment plans respectful of military		
			culture		

Acronyms: *ADL* activities of daily living, *ALL* anterior longitudinal ligament, *BPFS* back pain functional scale, *CAF* Canadian Armed Forces, *CAM* complementary and alternative medicine, *CFB* Canadian Forces Base, *CFHS* Canadian Forces Health Services, *CI* confidence interval, *CMT* chiropractic manipulative therapy, *CRP C* reactive protein, *DC* chiropractor, *DDD* degenerative disc disease, *DoD* Department of Defense, *ESR* erythrocyte sedimentation rate, *F/A* fighter/attack, *f/u* follow-up, *GMO* general medical officer, *HR* hazards ratio, *HVLA* SMT high velocity low amplitude spinal manipulative therapy, *LBP* low back pain, *MD* medical doctor, *mob*s mobilization, *MHS* military health system, *MSK* musculoskeletal, *MTF* military treatment facility, *NDI* neck disability index, *NHIS* National Health Interview Survey, *NRS* numerical pain rating scale, *OR* odds ratio, *PT* physical therapist, *RAAF* Royal Australian Air Force, *RMDQ* Roland-Morris Disability Questionnaire, *RTD* return to duty, *SD* standard deviation, *SE* standard error, *SMC* standard medical care, *SOF* special operation forces, *TBI* traumatic brain injury, *VPS* verbal pain scale, *UMC* usual medical care, *USMC* United States Marine Corps, *wks* weeks, *yo* years old, *yr*. years

### Location and access to chiropractic services

Six case reports [38–43], three randomized controlled trials [29, 30, 45], and one cross-sectional study [12] described chiropractic services provided to active duty military personnel worldwide. In North America, chiropractic services were reported as initiated through referral from a primary care provider (gatekeeper) following initial assessment, except in the randomized controlled studies where access was predetermined by study design [29, 30, 45].

As reported in the included articles, patients typically accessed chiropractic services through a gatekeeper, and were seen primarily for musculoskeletal conditions [16, 44]. The referral for chiropractic services may occur after the initial assessment, but most often occurred after initiation and non-response from other interventions [16, 41, 44]. Such other interventions included prescribed medication, diagnostic imaging, e.g. radiographs, magnetic resonance imaging, physical therapy, and referral to other healthcare services, e.g. pain clinic, specialist consultation. Direct access by active duty military personnel to chiropractic care was not reported in any included study.

Dunn et al. [16] in their qualitative study noted that chiropractic care was initiated into the US Department of Defense (DoD) in response to legislative action (1993–2009), and service has grown to 49 Military Health System commands. A survey to identify complementary and alternative medicine use at fourteen Military Treatment Facilities reported 92% of these facilities offered chiropractic services in 2005 compared to 85% in 2009 [34]. Additionally, the Military Health System Complementary and Alternative Medicine survey identified 55% of US Military Treatment Facilities offered chiropractic services in 2013 [31]. However, in 2005, 54% of active duty personnel resided in areas served by chiropractic clinics, with the remainder not served because of living overseas (14%), in remote areas (5%), or on bases with no chiropractic clinics (28%) [28].

In one Canadian study [12], chiropractic care was provided on-base in one location; however, this is no longer the case. Currently in Canada, chiropractic care is an eligible health benefit to CAF personnel and is accessed off-base, outside the military health system [44].

### Chiropractic scope of practice

The practice of chiropractic is the assessment of conditions related to the spine, nervous system, and joints and the diagnosis, prevention, and treatment of these conditions [﻿46﻿]. However, in Military Treatment Facilities, chiropractic scope of practice is established by the system/facility and may include comprehensive services (e.g. spinal manipulative therapy (SMT), mobilizations, modalities, rehabilitation), utilization of guidelines, and in some instances the performance of administrative tasks [16].

The chiropractic scope of practice described in the included articles consisted of both assessment and treatment. The chiropractic assessment included a focused history, physical examination, clinical impressions, disability, prognosis, and treatment plan elements. The interventions described in the articles included the following physical techniques and modalities: joint manipulation and mobilization of the spine and extremities [12, 29, 30, 38–42], soft tissue massage [12, 29], stretching/proprioceptive neurological facilitation maneuvers [12, 38–41], cryotherapy [30, 42], moist heat [42], superficial heat [30], McKenzie exercises [29], exercise [12, 29, 30, 38–42], interferential current [12, 30, 42], ultrasound [30], acupuncture [12], myofascial release [38], ischemic compression [38], advice on activities of daily living [29], postural/ergonomic advice [29, 39, 40], as well as nutritional and psychosocial aspects of treatment [42].

### Delivery model and benefits

In the US, active duty military personnel predominantly attended an on-base clinic [﻿16﻿, 29, 30, 38–43]. Care was described as interdisciplinary; however, healthcare providers typically provided services in physical isolation from other care providers. Case discussions occurred most often when prompted by a referral. In this healthcare model, the service member’s first point of contact is with a primary care provider who refers to chiropractic care if deemed necessary.

Chiropractors in the Military Health System are either contractors or employees of contractors, typically without healthcare and other benefits as part of their employment contract [16]. The chiropractor’s employment period is limited to the contract period. A decentralized leadership Military Health System structure may challenge the integration of chiropractic service into the Military Health System. Chiropractic care is a TRICARE benefit available to active duty service personnel but not their dependents [16].

### Challenges to collaboration

Challenges to collaboration were identified in two qualitative studies. Mior et al. described barriers to the integration of chiropractic services within the Canadian Forces Health Services (CFHS) [44]. Challenges to the integration of chiropractic services within the CFHS included base-to-base variation in referral procedures, which were associated with clinician preference and experience rather than a systematic approach. Mior et al. also reported that inter-professional communication varied by base and typically lacked standardized reporting [44]. The minimal reported interaction between chiropractors and CFHS healthcare providers apparently impeded the development of a positive inter-professional relationship. Chiropractic care was perceived as a duplication of physical therapy services, often considered more as a single intervention, that is spinal manipulative therapy, rather than as a profession.

Dunn et al. [16] identified that untimely access, unavailable services, and unobtained referrals could affect the integration of chiropractic services in the Military Health System. Despite the legislative mandates in the US, Dunn et al. argued that continued acceptance and integration will depend upon the chiropractors adding measurable value to service delivery [16].

Both qualitative reports suggested that improved collaboration and/or integration was not possible without service availability at military treatment facilities. Also, ensuring care provided was consistent with the needs of the patients, while being respectful of the roles and responsibilities of others, was argued as important to ensure sustainable integration [16, 44].

### Opportunities

Opportunities to include chiropractic services within the active duty military were identified in several articles [16, 31, 44]. These opportunities included providing clinical, cost effective evidence-based care for musculoskeletal conditions within an environment of inter-professional collaborative care. Specifically, within this environment the delivery of care would be based on clinical practice guidelines that draw upon the full scope of chiropractors’ practice rather than solely the delivery of a specific intervention.

### Utilization of chiropractic services

The utilization rate of chiropractic services by active duty military members was reported in seven cross-sectional surveys [28, 32–37]. The reported utilization rate of chiropractic services over the preceding 12 months was consistent over time (2000–2011) in the United States but then decreased in 2013. Specifically, the utilization rate ranged between 5.2 and 10.5% among active duty military personnel [28, 32, 35–37] and 14.8% in the Reserve/National Guard in 2007 [35], to a low of 2.9% in 2013 among services in the Military Health System [49]. In Australian military aircrew, 12% sought chiropractic services for flight-related neck pain [33], which is higher than the 12-month chiropractic service utilization in the US in the period 2008-2011.

### Type of conditions treated and duration of treatment period

Back pain with or without radiculopathy accounted for the majority of presentations [12, 29, 30, 38, 39]. In a US cross-sectional study, 42.7% of respondents reported using chiropractic services for low back pain (LBP), 27.3% for headaches, and 10.9% for general health, wellness, and prevention [31]. In a Canadian cross-sectional survey, 97% of patients reported spine-related musculoskeletal complaints, of which 52% were LBP, and 3% involved conditions of the extremities [12].

Neck pain was reported as the reason for chiropractic consult in several articles [12, 33, 40, 41, 43]. In a survey of Australian air force personnel, 12% of the respondents sought chiropractic care for neck pain [33]. Neck pain with radiating symptoms was reported in one cross-sectional study [﻿12﻿]. Aside from one of the included randomized controlled trials [29], most articles reported patients presenting with chronic musculoskeletal conditions [12, 30, 31, 40, 43].

Chiropractic treatment duration was reported in nine articles and varied considerably between articles [12, 29, 30, 38–43]. In their cross-sectional study, Boudreau et al. [12] reported the average number of chiropractic visits as 5.7 ± 4.1 (mean ± SD), ranging from one to 25 visits. Goertz et al. [29] in their randomized control trial (RCT) scheduled up to two chiropractic visits weekly (eight visits) for a period of 4 weeks, and reported participants attended an average of seven visits. In another randomized controlled trial, patients were allocated to up to 12 visits over 6 weeks [30], with patients utilizing a much smaller number than the available number of visits. Patients who attended at least one visit to a chiropractor reported a mean number of chiropractic visits (mean ± SD) which varied across study sites and ranged from 2.3 ± 1.4 (San Diego), 4.7 ± 2.5 (Walter Reed), to 5.4 ± 2.6 (Pensacola) [30]. In a survey of US Military Treatment Facilities offering chiropractic services, the average number of patient visits to a chiropractor was 5.4, the highest reported patient visits of any complementary and alternative medicine provider in 2013 [31].

Case report participants reported the greatest number of chiropractic visits. The number varied from 16 visits over 30 weeks with an aviator instructor with acute LBP [38], a military officer with C3–5 anterior longitudinal ligament heterotopic ossification and ankyloses received 34 visits over 47 weeks [43], and a Naval Petty Officer with low back and radicular pain attended 11 visits over 72 days [42].

### Outcomes of care

Reported outcomes of chiropractic care were predominantly positive. Favourable outcomes following chiropractic care were reported in each of the case reports, however in one case report the patient improved but did not return to duty [43]. In a Canadian cross-sectional study, active duty military reported satisfaction with care (94.2%) [12]. Further, all physicians in this study identified a perceived demand for chiropractic services, and the majority (80.6%) were satisfied with chiropractic services [12].

In a RCT, Goertz et al. [29] reported a mean difference favouring chiropractic manipulative therapy (CMT) in addition to standard medical care (SMC) over SMC alone for each of the primary outcomes at 2 and 4 weeks in acute LBP subjects. A greater percentage of participants in the SMC plus CMT group (73%) rated their global improvement as pain completely gone, much better, or moderately better, compared with 17% in the SMC group. Similarly in another RCT, Goertz et al. [30] reported mean differences favouring usual medical care (UMC) with chiropractic care (CC) over UMC alone for each of the primary outcomes at 6 and 12 weeks, although the magnitude of difference decreased at 12 weeks. Additionally, secondary outcomes of worst LBP intensity and symptom bothersomeness also favoured UMC + CC over UMC. Overall, UMC + CC identified better global perceived improvement, satisfaction with care, and used less pain medication.

The DeVocht et al. [45] RCT assessed if a short course (4 visits) of CMT improved reaction and response time outcomes in special operation forces military compared to wait-list control. Despite observing an immediate effect after the first session on complex response task, no significant between group differences were reported for any of the outcome measures at 2 weeks.

### Adverse events

Adverse events were reported in three articles, all RCTs [29, 30, 45]. Although there were no serious adverse events reported, two studies reported 6 minor events of which 5 were unrelated to trial procedures and 1 related to SMT [45]. In a large RCT [33], there were 62 events reported, where 19 were in the usual care group and 43 in the usual care and chiropractic care group. The majority (49/62) were reported as muscle or joint stiffness related to either chiropractic care, physiotherapy care or self-care.

## Discussion

We found 20 articles that described chiropractic services within the active military worldwide. The majority of articles (*n* = 17) were from the US, with additional information provided from articles from Canada (*n* = 2) and Australia (*n* = 1). The majority were cross-sectional studies assessing utilization or access of chiropractic services or case reports highlighting common or unique conditions managed; there were only three RCTs evaluating chiropractic as an intervention in this setting. The majority of included articles were published between 2006 and 2010 (*n* = 9) but the more robust designed clinical studies were published in the last 2 years. There is less research related to chiropractic services in active military personnel compared to that involving veterans [16, 47].

We found that chiropractic services are provided to active duty military in on-base clinical facilities in only one country, the US; this is driven by US legislation [16]. Regardless of location, services are typically accessed through a gatekeeper, usually a medical physician [16, 44]. Care delivery models vary but the extent of integration of chiropractic services within the US Military Health System remains unclear. In the US and Canada, chiropractic services are available to active duty military; however, they are delivered by paid contracted chiropractors in the US, as opposed to a third party insured military personnel benefit in Canada. It is unclear what service delivery models exist in countries other than the US and Canada as we located no articles describing this. However, it appears that the inherent gatekeeper referral processes influence the access to chiropractic services.

Reported 12-month utilization of chiropractic services in the US ranged from 2.9 to 10.5% between 2000 and 2013 [28, 32, 36, 37]. Outside of the US, we found only one study reporting a 12% utilization of chiropractic services among Australian military aircrew [33]. The reported utilization seems similar to that reported in the general population [48].

Aside from one randomized controlled trial which included acute LBP patients, most articles reported patients presenting with chronic musculoskeletal conditions. These findings are similar to those seen in the general population, where musculoskeletal conditions are the predominant reason for consulting chiropractors [48]. The frequency of chiropractic visits reported in cross-sectional studies ranged from a mean of 5.4 in a survey of US Military Treatment Facilities [31] to 5.7 in a single Canadian base [12]. In Canada, limits to covered benefits and policy may influence visit frequency.

Outcomes of care provided by chiropractors was positive in most of the reported clinical studies; however, six of these were case report designs that cannot evaluate effectiveness. In two included RCTs, outcomes favoured usual medical care and chiropractic care compared to standard medical care alone. These findings are consistent with recent LBP guidelines supporting the use of manual and conservative care [49–51]. However, in the larger LBP pragmatic trial [30], treatment included interventions of questionable effectiveness [51]; suggesting further clinical trial data are required to assess if practice is consistent with current guidelines.

In another RCT, the use of a short course (4 visits over 2 weeks) of chiropractic manual therapy to improve select measures of performance in special operations military personnel was no different from wait-list control [45]. Despite empirical evidence of performance enhancement following manual therapy, the immediate but not statistically significant longer-term effect reported in this study is consistent with findings in systematic reviews assessing the impact of manual therapy on performance [52, 53].

Our review adds to a previous review examining the integration of chiropractic services in military and veteran health care facilities [47]. We add new information assessing chiropractic services in active duty military from articles in the US, Canada, and Australia. Unfortunately, we found no evidence of chiropractic services provided to active duty military in other countries. The majority of the articles emanated from the US where chiropractic services were included in the Military Health System since 1995 [47]. Little is known about the nature of integration of chiropractic services in the US Military Health System, but evidence suggests that it varies from base-to-base [16, 47]. We add new information from Canada highlighting the challenges and opportunities of the inclusion of chiropractic services in active duty military [44]. Given utilization data is limited to the US and Australian Air Force, further descriptive studies are required to fill this gap worldwide.

Understanding the characteristics of chiropractic services provided is important in assessing and maximizing quality of care [54]. The significant expansion of chiropractic services within the US Military Health System has been largely driven by legislative directives, which in themselves may challenge the nature and extent of system integration [16]. If value of services is measured by system needs rather than that of the providers [16], then system and care-based outcomes are important assessment metrics required to ensure continued success. Our scoping review suggests little is known about the clinical and quality metrics of chiropractic services in active duty military globally. Qualitative studies could provide the necessary understanding of the system and resource barriers and potential opportunities for inclusion or expansion of chiropractic services worldwide.

### Strengths and limitations

A strength of our scoping review was the systematic process used to collect and summarize the evidence from this diverse body of literature. A scoping review is the most appropriate method to collect and organize diverse information and to develop a picture of the existing evidence base when a broad research question is asked [55]. Our health sciences librarian conducted a broad and methodologically rigorous literature search, which was reviewed by a second librarian. Further, we searched two military specific databases in an effort to capture all discipline specific relevant articles. Study selection was based upon detailed inclusion and exclusion criteria to ensure that consensus between paired independent reviewers was transparent and reproducible.

There are limitations in this review. In keeping with the scoping review framework we collated the evidence on chiropractic care in the military and did not critically appraise the methodology of the reported articles [19]. Future systematic reviews focusing on the specific factors discussed in this review should include an appraisal of the study methods. We restricted our search to include articles in the English language, which may have excluded some relevant articles. However, chiropractic journals publish in English, which is recognized as the standard language of science, thereby reducing this risk [56].

## Conclusion

Our scoping review explored the available evidence related to chiropractic services within active duty military. The majority of the articles emanated from the US and were cross-sectional in nature. Two recent RCTs provide evidence of comparative effectiveness of adding chiropractic care to usual medical care. Despite the reported use of chiropractic services in Australia, Canada, and the US, there is little available published evidence related to the nature, use, and outcomes of chiropractic care in active duty military. Our review suggests the need for further qualitative, descriptive, and clinical trial data worldwide to inform the role and value of chiropractic services in active duty military globally.

## Additional files

Additional file 1: Search Strategy (DOCX 21 kb)

## Data Availability

Not applicable.
