# Supplementary material for: Chiropractic services in the active duty military setting: a scoping review
Source: Chiropr Man Therap. 2019 Jul 15;27:45. doi: 10.1186/s12998-019-0259-6 (PMC6628474; doi:10.1186/s12998-019-0259-6)
Supplement: Supplementary file 1 — Search Strategy (DOCX 21 kb) [file 12998_2019_259_MOESM1_ESM.docx]

**Additional file 1:** Search Strategy

| **#** | **Searches** |
| --- | --- |
| 1 | Aviation Medicine/ |
| 2 | Hospitals, Military/ |
| 3 | Military Facilities/ |
| 4 | Military Medicine/ |
| 5 | Military Personnel/ |
| 6 | United States Department of Defense/ |
| 7 | Warfare/ |
| 8 | (active adj duty).ab,kf,ti. |
| 9 | (active adj service).ab,kf,ti. |
| 10 | (air adj force*).ab,kf,ti. |
| 11 | (American adj2 force*).ab,kf,ti. |
| 12 | (armed adj force*).ab,kf,ti. |
| 13 | (army or armies).ab,kf,ti. |
| 14 | "battalion*".ab,kf,ti. |
| 15 | (Canadian adj2 force*).ab,kf,ti. |
| 16 | (coast adj guard*).ab,kf,ti. |
| 17 | "conscript*".ab,kf,ti. |
| 18 | "defence force*".ab,kf,ti. |
| 19 | (Defence adj Department*).ab,kf,ti. |
| 20 | (Defense adj Department*).ab,kf,ti. |
| 21 | "defense medical service*".ab,kf,ti. |
| 22 | "defense health agenc*".ab,kf,ti. |
| 23 | (Department adj Defense).ab,kf,ti. |
| 24 | (Department adj Defence).ab,kf,ti. |
| 25 | (force* adj2 (defence or defense)).ab,kf,ti. |
| 26 | (marine adj2 corps).ab,kf,ti. |
| 27 | marines.ab,kf,ti. |
| 28 | (militar* adj5 (healthcare or health) adj (care or installation* or medicine or personnel or population* or service or facilit*)).ab,kf,ti. |
| 29 | (national adj guard).ab,kf,ti. |
| 30 | ((navy or navies or naval) adj5 (healthcare or health) adj (care or installation* or medicine or personnel or population* or service or facilit*)).ab,kf,ti. |
| 31 | "reservist*".ab,kf,ti. |
| 32 | "sailor*".ab,kf,ti. |
| 33 | "soldier*".ab,kf,ti. |
| 34 | "submariner*".ab,kf,ti. |
| 35 | or/1-34 [**military] |
| 36 | Chiropractic/ |
| 37 | Complementary Therapies/ |
| 38 | Manipulation, Chiropractic/ |
| 39 | Manipulation, Spinal/ |
| 40 | Musculoskeletal Manipulations/ |
| 41 | "chiropract*".ab,kf,ti. |
| 42 | (alternative adj2 (care or health or medicine* or practitioner*)).ab,kf,ti. |
| 43 | (complementary adj2 (care or health or medicine* or practitioner* or therap*)).ab,kf,ti. |
| 44 | (manipulat* adj3 (cervical or lumbar or musculoskeletal or spinal or spine or thoracic)).ab,kf,ti. |
| 45 | (non-conventional adj2 (health or practitioner* or therap* or medicine*)).ab,kf,ti. |
| 46 | (secondary adj2 (care or health or medicine* or practitioner* or therap*)).ab,kf,ti. |
| 47 | (unconventional adj2 (health or practitioner* or therap* or medicine*)).ab,kf,ti. |
| 48 | or/36-47 [**chiropractic] |
| 49 | 35 and 48 |
| 50 | limit 49 to english language |
